# Hubble Bubble Equals Trouble: The Hazards of Water Pipe Smoking

**DOI:** 10.1100/tsw.2006.332

**Published:** 2006-02-02

**Authors:** Jacob Urkin, Rivka Ochaion, Aya Peleg

**Affiliations:** ^1^ Primary Pediatric Unit, Faculty of Health Sciences, Ben-Gurion University of theNegev, Beer-Sheva, Israel; ^2^ Clalit Health Services, Beer-Sheva, Israel

**Keywords:** Narghile, water pipe, tobacco, adolescents, substance abuse, Israel

## Abstract

A Narghile, one of the names for a water pipe, is an instrument for tobacco smoking that has became a trend among the youth in Israel. The mistaken opinion that smoking a Narghile is not as dangerous as smoking cigarettes makes the youngsters and their parents take it lightly and contributes to the expansion of its use. The objective of this paper was to review the literature on the health risks of Narghile smoking. A literature search of Medline (PubMed) and the Internet on the health hazards of Narghile smoking was conducted. The health hazards that the Narghile smoker is exposed to include interference with oxidation, damage to genetic compounds, increased risk of developing malignancies, infectious diseases, damage to the fetus and newborn, and exposure to commonly abused chemicals. It is concluded that increased awareness of the expanded use of the Narghile is imperative and education programs about the prevention of cigarette smoking and substance abuse should also include Narghile smoking.

## INTRODUCTION

During recent years, smoking a Narghile (water pipe) has become a trend among Israeli youth[1]; 15% of seventh graders and 58% of ninth and eleventh graders smoked a Narghile at least once a week. Among the adolescents who smoked using a Narghile, 52% acknowledged that their parents knew about that habit and 23% had experienced cosmoking with a parent[1]. Only 35% of the parents of tenth-grade students objected to Narghile smoking. These findings are of major concern[1,2].

The reasons for preferring Narghile smoking as reported by the teenagers were the lack of damage caused by Narghile smoking, a belief that the harmful substances in the tobacco were filtered by the water container of the instrument, that the Narghile smoking had a pleasant feeling of moisture in contrast to the burning sense of cigarette smoking, lack of parental objection, that Narghile provided the sense of social belonging, that Narghile smoking is perceived as a leisure activity, and availability in the teenagers' places of entertainment.

A recent review by Knishkowy and Amitai[2] described the myths, misconceptions, and facts regarding water-pipe smoking. Some youngsters think that the total harm from Narghile smoking is less than that from cigarette smoking because it is used more episodically. They also tend to ignore the potential danger of drugs that can easily be added to a Narghile. Narghile smoking is a social activity and a hobby. Youngsters collect Narghiles, they experience smoking different kinds of tobacco, prepare recipes, and experience different methods of smoking. Possessing a personal Narghile is very common and, in many cases, smoking a Narghile starts earlier than smoking cigarettes and thus is a gateway to cigarette smoking.

### The Structure and Function of the Narghile

The Narghile is a tobacco-smoking instrument. It is comprised of a bowl at the top, a body, a pipe with a mouthpiece, and a glass bottle at the base of the instrument to hold the water. Tobacco is burned in the top bowl of the Narghile by burning coal embers next to the tobacco. Suction through the mouthpiece draws the smoke through the water in the glass bottle and then into the mouth and lungs of the smoker.

### Historic Background

The Narghile has many names. In English, it is called “Hubble Bubble” or “Water Pipe”. In Turkey, the “Hookah”; in Egypt, “Shisha” or “Goza”; and in Lebanon, it is called "Narghile”. Smoking a Narghile is a central part of the coffee-shop culture in the Middle East. In the first half of the 20th century, it was used in Turkey and rules for its lighting were created. Simultaneous with the increase of cigarette consumption, Narghile use dropped to a point that it was used almost exclusively by elderly people in the poor areas of Arabia. In the last decades, in spite of the health education programs that warn against its use, there is a rise in Narghile smoking all over the Middle East.

In Muslim society, smoking a Narghile is perceived differently than cigarette smoking. Out of respect, a young male should not smoke a cigarette in front of his parents and a female should not smoke in front of males. In contrast, Narghile smoking is not perceived as a custom that shows disrespect. It is socially accepted that a father may offer the Narghile to his adolescent child. In addition, cigarette smoking is associated with tense and busy people, whereas Narghile smoking is associated with leisure.

The Narghile is commonly used in coffee shops and tourist sites in the Middle East, thus, tourists from other parts of the world are exposed to it. Some bring a Narghile to their home country and continue to use it. Emigration of people from the Middle East to other parts of the world also contributes to the expansion of Narghile use. Few studies have challenged the health effects of Narghile smoking[2]. The purpose of this article was to summarize the hazards of Narghile smoking based on the type of adverse effects.

## METHODS

The objective was to review the literature on the health risks of Narghile smoking. A literature search of Medline/PubMed (http://www.ncbi.nlm.nih.gov/entrez/query.fcgi?DB=pubmed) and the Internet on the health hazards of Narghile smoking was conducted**.** The search was not limited to recent studies as the number of medical publications on Narghile smoking were relatively limited. The keywords for search included water-pipe smoking and the alternative names common in different countries to describe the smoking device. The references cited were chosen based on their relevance to the risk of Narghile smoking. These studies are of various design.

## EXPOSURE TO HARMFUL COMPOUNDS

Contrary to common impressions, the Narghile does not protect smokers from the tar in the smoke. Gieringer[3] summarized the efficiency of various smoking instruments in decreasing the concentration of tar. He concluded that in comparison with cigarette smoke, Narghile smoke contains an increased concentration of tar and volatile carcinogens. This is supported by a recent study on the composition of the smoke aerosol of the Narghile water pipe[4].

Narghile smokers are also exposed to carbon monoxide (CO), which is generated by incomplete burning of carbon fuels. By combining with hemoglobin, carboxyhemoglobin (COHB) is formed, reducing the oxygen-carrying capacity of the blood. The size of a Narghile influences the concentration of CO in the smoke. The smaller the Narghile and its burning bowl, the lower the air volume in its water base and the shorter the length of the inhalation tube, correlating with higher concentrations of CO. A three- to fourfold increase of CO concentration was demonstrated when the smoke of cigarettes was compared to that of large Narghiles ($p < 0.01$). Dissolved CO in the plasma penetrates body cells and paralyzes intracellular oxidation, thus harming the function of essential organs such as the heart and the brain. Continued exposure to high levels of CO can increase the development of lung diseases such as chronic bronchitis. Burning of dry tobacco produced more CO than did humid tobacco ($p < 0.05$). The use of industrial coal, made of hard wood, results in higher levels of CO when compared to homemade coal, made of soft wood[5].

Zahran et al.[6] have demonstrated a high percentage of COHB in a Narghile smokers' group (8.8 ± 1.83) compared to the cigarette smokers' group (6.1 ± 2.58) or to controls who did not smoke (1.7 ± 0.55) ($p < 0.001$). In addition, symptoms indicating CO poisoning, such as blurred vision, rapid pulse, and dizziness were more frequent in Narghile smokers than in the cigarette smokers[6]. Support for the above findings was obtained in the research done by Sing et al.[7] in India. Levels of COHB and blood cholesterol of smokers of various tobacco types such as cigarettes and Narghiles were compared. The highest levels of COHB were found among those who had smoked a Narghile for more than 10 years ($p < 0.001$)[7]. Even a single hubble bubble run resulted in increased end-expiratory CO[8].

Shafagoj et al.[9] of Amman University have demonstrated that following Narghile smoking, high nicotine and cotinine levels are sustained for prolonged periods of time. The conclusion of their study was that Narghile smoking may lead to addiction to nicotine and it is not a reasonable substitute for cigarettes[9].

The main stream of the Narghile smoke contained significant amounts of nicotine, tar, arsenic, chromium, and lead[10]. In research conducted by Sukumar et al.[11] of Delhi University, the hair and fingernails of subjects were examined for trace elements including Cadmium (Cd), Chromium (Cr), Copper (Cu), Manganese (Mn), Nickel (Ni), Lead (Pb), and Zinc (Zn). These elements were found in cigarette and Narghile smoke. High levels of Cd were found in the hair of both genders who smoked tobacco in general, and Narghiles in particular, when compared to a group of nonsmokers ($p < 0.05$). Cd is a carcinogen[12].

As a smoking instrument, the Narghile can be used with cannabis and marijuana in addition to tobacco. Adding or using alcoholic beverages in the water bottle of the Narghile adds to the risks of substance abuse[1,2].

## LUNGS, CARDIOVASCULAR FUNCTIONS, AND BLOOD

Kiter et al.[13] examined the influence of Narghile smoking on pulmonary function. They compared lung function of subjects in four groups: exclusive Narghile smokers, present Narghile smokers who smoked cigarettes in the past, cigarettes smokers, and a control group of nonsmokers. The study demonstrated a reduction in lung function tests among all the smokers.

Similar results were demonstrated by Al-Fayez et al.[14] who examined the influence of cigarette and Narghile smoking on lung function in males and females. A decrease in lung function tests was observed in males aged 20–49 years who had smoked Narghiles. Lung function tests of females aged 20–49 years smoking Narghiles were worse than those of older women who used to smoke cigarettes.

Neutrophils of smokers have higher levels of superoxide, when compared to nonsmokers ($p < 0.001$). Leukocytes of Narghile smokers have higher levels of superoxide than those of cigarette smokers ($p < 0.05$)[15]. It was assumed that obstructive lung disease is caused, in part, by the release of superoxide from leukocytes in the lungs.

Acute short-term active hubble-bubble smoking elicits a modest increase in heart rate, systolic blood pressure, diastolic blood pressure, and mean arterial blood pressure[8].

Wolfram et al.[16] examined the impact of Narghile smoking on the oxidative stress of blood platelets and on their function. Volunteers who were nonsmokers were asked to smoke a Narghile daily for 14 days. Evaluations of platelets were done before, immediately after, and 6 h after smoking, and repeated 1 and 2 weeks afterwards. Long-lasting oxidation injury to platelet function was demonstrated following Narghile smoking[16].

## NARGHILE AND INFECTIOUS DISEASE

By sharing the mouthpiece of the pipe, bacteria, viruses, and fungi can pass between Narghile smokers. A report from Tunisia mentioned the potential existence of mycobacteria in the Narghile tube as a source of contamination of different customers[17].

According to the report by Szyper-Kravitz et al.[18], *Aspergillus pneumonia* in a patient who had leukemia was related to Narghile smoking. The patient reported daily smoking of a Narghile with marijuana. Cultures of the Narghile's tobacco found that the humid tobacco was contaminated with *Aspergillus* spores, which cause the pneumonia. The risk of contracting *A. pneumonia* is higher in immunocompromised people.

In an attempt to identify the risk factors of the Hepatitis C virus (HCV) in Egypt, Habib et al.[19] conducted a study in which they found that, in addition to sharing a razor by the local barber, there was an additional risk factor for Hepatitis C based on sharing a Narghile mouthpiece. In both circumstances, transmission of the disease occurred by infectious blood drops that clung to the tool. Widespread gingival infections in Egypt make this route of infection likely. *Helicobacter pylori* may also pass via a shared mouthpiece among smokers. El-Barraway et al.[20] have demonstrated that the risk of *H. pylori* infection was significantly higher among moderate to heavy smokers of cigarettes and among smokers who shared Narghiles. Derivatives of tobacco smoke that enter the body can reduce the resistance of the stomach mucosa to its acidity and thus increase the risk of developing an ulcer.

The relative risk of periodontal disease is 5.1-fold increased in water-pipe smokers compared to nonsmokers ($p < 0.01$)[21].

## MALIGNANT DISEASE

During the process of tobacco burning, pyrolysis, hydrogenation, decarboxylation, and dehydration take place. Thousands of chemicals, some of them harmful, are generated by incomplete burning. Compounds that bond to nucleic acids are potentially carcinogenic. Tobacco is the most important risk factor for oral cancer[22]. El-Hakim et al.[23] have found a positive correlation between lip, cheek, and mouth cancers (squamous cell carcinoma and keratoacanthoma) and Narghile smoking. In their research, they noted that Narghile tobacco contains a juicy compound that causes chronic irritation, increasing vulnerability to developing malignant diseases of the mouth.

In a case control study by Lubin et al.[24], the exposure to radon and its influence on the occurrence of lung cancer was evaluated. Subjects with more years of Narghile smoking had a greater risk for lung cancer. Research in India by Gupta et al.[25], found that the attributed risk for lung cancer among Narghile smokers was identical to the risk among cigarette smokers. Similar results were published by

Lubin et al.[26] who found that the highest risk was in tobacco smokers who were using the Narghile and also smoked cigarettes. Gunaid et al.[27] performed case control research among 3,064 patients who, for whatever reason, had an endoscopy. After adjustment for demographic and social data and various smoking habits, a high risk for tumors of the gastroesophageal junction and cardia was found among persons who smoked a Narghile and in those who were habitual Gath chewers.

## FERTILITY, FETUS, AND GENETICS

Maziak et al.[28] have recently noted the increasing popularity of Narghile smoking in developing regions in Asia and the Middle East. Their review describes pregnancy-related complications and calls for more research on its health effects. Low birth weight, lower Apgar scores, and neonatal respiratory distress were related to Narghile smoking during the first trimester of pregnancy[29]. Research in Egypt indicated an association between Narghile smoking and fertility problems[30].

The influence of Narghile smoke on genetic material was investigated by Yadav and Thakur from Kurukashetra University in India. Mitosis index (MI), chromosomal aberration (CA), sister chromatid exchange (SCE), and deviation of the satellite structure of the chromosomes were associated with Narghile smoking[31].

## CONCLUSIONS

Smoking a Narghile involves many health risks (see Table 1). There are a limited number of studies concerning Narghile smoking compared with those on cigarette smoking.

The Narghile is a common device that contributes to the addition of large numbers of teenagers to the smoking population in many countries. It is a gateway to the world of cigarette and substance abuse. Lack of surveys in western countries on this matter call for attention. A Narghile is easy to use. It has the charm of the oriental state of mind and can easily be used with chemicals that are not tobacco. Public awareness is needed to find early and effective ways to reduce the spread of its use. Beginning Narghile smoking at a younger age than that for cigarettes and false ideas that it is less harmful, challenge educators and health professionals. Curiosity, rebellion, social pressure, and risk-taking behavior play important roles in turning young people to use the Narghile. For them, it is one of many ways to gain new experiences, to explore limits, and to express their own will, in spite of the risk involved. Unfortunately, the risks are not well known to health professionals, educators, and parents. In order to reduce Narghile smoking, action should take place through different interventions. The media should be used to convey the message that smoking a Narghile is at least as dangerous as smoking cigarettes. As it is not acceptable to sell alcoholic beverages and cigarettes to minors, providing opportunities for minors to smoke a Narghile should also be outlawed. Parents should be made aware that smoking a Narghile is harmful and acceptance of Narghile smoking by their child is an act of neglect. Educators and health care professionals should be more vigilant in bringing the information to children and public. They should serve as good role models and be active in the process of appropriate legislative efforts to limit the use of Narghiles. Public health authorities should lead the struggle against Narghile smoking as the impact of its use is expected to affect the health and life of many. Research should focus on the behavioral aspects of the young population who are exposed to Narghiles. Behavioral modification techniques, change in cultural attitudes toward Narghile use, and legislative actions should be applied in concert to find the way to the hearts and minds of Narghile users. Development of effective ways for prevention and early intervention are crucial.

**TABLE 1**
**Adverse Effects from Narghile Smoking**

| | Mechanism | References |
|---|---|---|
| **Type of Damage** | | |
| Impaired oxidation | High plasma levels of COHB and of CO | 5,6,7 |
| Damage to lung tissue | Increase in number of neutrophils with superoxide; iflammation of airways | 13,14 |
| Increased blood pressure | | 8 |
| Addiction to tobacco and other substances | Exposure to nicotine, cotinine, and added chemicals | 1,9 |
| Impact of toxic elements | Heavy metals | 11,12 |
| Impaired platelet functions | | 16 |
| **Infectious and Malignant Diseases** | | |
| Tuberculosis | Transmission through shared mouthpiece | 17 |
| Hepatitis C virus | Transmission through viral-containing blood drop in shared mouthpiece | 19 |
| *A. pneumonia* | Tobacco contaminated with fungus | 18 |
| Gastric ulcer | Transmission of *H. pylori* through shared mouthpiece | 20 |
| Perodontal disease | | 21 |
| Malignant diseases: squamous cell carcinoma, keratoacanthoma, lung cancer, esophageal-stomach cancer | Exposure to carcinogens secondary to incomplete burning of tobacco | 22,23,24,25,26,27 |
| **Impact on Fetus, Newborn, and Fertility** | | |
| Low birth weight and infertility; perinatal morbidity and mortality | Disruption of metabolism between the fetus' blood and the mother's blood; exposure of the fetus to the toxic chemicals in tobacco smoke | 29,30 |
| Damage to male and female fertility and to genetic compounds | Exposure to mutagens | 31 |

## BIOSKETCHES

**Jacob Urkin MD, MPH**, is a lecturer in pediatrics, Director of the Primary Pediatric Unit, and Director of the Maternal and Child Program of the MPH program at the Faculty of Health Sciences, Ben-Gurion University of the Negev, Beer-Sheva, Israel. Major academic interests are primary care, community pediatrics, well-baby care services, school health, and medical education. E-mail: jacobur@clalit.org.il

**Rivka Ochaion RN, MPH**, is a general and well-baby care nurse in a rural community clinic and a PhD student in public health at the Faculty of Health Sciences, Ben-Gurion University of the Negev, Beer-Sheva, Israel. Major academic interest is the epidemiology of malignant disorders. E-mail: krispel@bgu.ac.il

**Aya Peleg, PhD**, is a lecturer in epidemiology, Department of Epidemiology, at the Faculty of Health Sciences, Ben-Gurion University of the Negev, Beer-Sheva, Israel. Major academic interests are substance abuse, smoking cessation, and health promotion. E-mail: ayapeleg@bgu.ac.il
